# Impact of climatic oscillations on marlin catch rates of Taiwanese long-line vessels in the Indian Ocean

**DOI:** 10.1038/s41598-023-49984-4

**Published:** 2023-12-17

**Authors:** Sandipan Mondal, Aratrika Ray, Kennedy Edeye Osuka, Riah Irawati Sihombing, Ming-An Lee, Yu‑Kai Chen

**Affiliations:** 1 Department of Environmental Biology and Fishery Science, National Taiwan Ocean University, Keelung City, 202 Taiwan; 2 Center of Excellence for the Oceans, National Taiwan Ocean University, Keelung City, 202 Taiwan; 3 Department of Earth, Ocean and Ecological Sciences, University of Liverpool, Liverpool, L69 3BX UK; 4 Executive Yuan, Coastal and Offshore Resources Research Center of Fisheries Research Institute Council of Agriculture, Kaohsiung, 80672 Taiwan

**Keywords:** Environmental sciences, Ocean sciences

## Abstract

This study explored the influence of climatic oscillations on the striped, blue, and silver marlin catch rates in the Indian Ocean by using logbook data from Taiwanese large-scale fishing vessels and climate records from 1994 to 2016. Only the Madden–Julian oscillation (MJO) and the subtropical Indian Ocean dipole (SIOD) had immediate effects on the striped and silver marlin catch rates. The positive and negative phases of the IOD at the lags of 7 and 3 years corresponded to increased and decreased catch rates, respectively, for both the striped and blue marlin, contrasting to the reverse pattern for the silver marlin. Similarly, all three marlin species experienced decreased and increased catch rates respectively during the positive and negative phases of the Pacific decadal oscillation. The striped and blue marlin catch rates decreased and increased during the positive and negative phases, respectively, of the SIOD and MJO with various lags. Our results suggest that the impacts of climatic oscillations on fish species are crucial for policymakers and coastal communities for managing marine resources, forecasting changes in marine ecosystems, and developing strategies to adapt to and mitigate the effects of climate variability.

## Introduction

Extreme ocean events are significantly influenced by the frequency, intensity, and behavior of ocean conditions^1^. Changes in ocean conditions can cause extreme ocean phenomena such as hurricanes, cyclones, storm surges, marine heatwaves, and coastal inundation. An increase in sea surface temperature (SST) can trigger the development and intensification of these events by providing the necessary energy. This process can lead to potentially more frequent extreme ocean events^2^. Moreover, alterations in ocean circulation patterns can affect the paths or behaviors of such events. For example, the intensity and location of the Gulf Stream in the North Atlantic can affect the course of hurricanes^3^. Thus, fluctuations in ocean conditions substantially affect extreme ocean events.

Climatic oscillations, also referred to as climate cycles or variations, exert a profound effect on ocean conditions and dynamics^4^. These oscillations are natural patterns that recur on timescales ranging from years to millennia. They affect various ocean conditions, including SST, ocean currents, precipitation patterns, and sea level^5^. For instance, during the negative phase (El Niño) of El Niño-southern oscillations (ENSO), the tropical Pacific can lead to substantial SST rises, causing widespread warming, whereas the positive phase (La Niña) have the opposite effect^6^. The Pacific decadal oscillation (PDO) affects the strength and direction of key ocean currents such as the Gulf Stream^7^, and the ENSO affects precipitation patterns in Australia and other regions in the tropical Pacific^8^. The effects of these climatic oscillations on ocean conditions are complex and can vary with the oscillation's phase and strength and interactions with other climate factors. Moreover, climatic oscillations can exert delayed impacts on ocean conditions because their anomalies persist for months and years following the events. Despite studies demonstrating that lagged climatic oscillations are related to current ocean conditions^9,10^, studies on the effect of climatic oscillations on migratory pelagic species in the Indian Ocean remain sparse.

The effect of climatic oscillations on marine ecosystem is profound^11^. For instance, during El Niño events, the warming of the eastern Pacific Ocean disrupts the food chain and affects fish populations^12^. Changes in SST influence water column stability, leading to a shallower mixed layer when the SST is warmer than the water beneath it. This inhibits vertical mixing and reduces the likelihood and intensity of upwelling in regions with a shallow mixed layer, thereby decreasing productivity and disrupting the distribution of marine communities^13^. Extreme ocean events and changes in ocean circulation also alter the distribution of plankton and other food sources^14^.

Climatic oscillations in the Indian ocean affect regional weather patterns, oceanic conditions, and ecosystems. The Indian Ocean dipole (IOD) is a climatic oscillation characterized by variations in Indian Ocean SST^15^. Other climatic oscillations of the Indian Ocean include the Madden–Julian oscillation (MJO) and Subtropical Indian Ocean Dipole (SIOD)^16^. The MJO is an eastward-moving cloud, precipitation, and wind disturbance that primarily affects the Indian Ocean^17^. The SIOD is characterized by anomalies in subtropical Indian Ocean SST^18^. Each of these oscillations uniquely affects fisheries in the Indian Ocean^19^. For example, during positive IOD phases, higher SST in the western Indian Ocean and lower SST in the eastern Indian Ocean near Indonesia create a temperature gradient that influences ocean and atmospheric circulation patterns. These alterations in ocean conditions affect fish catches. Gaol et al.^20^ reported a decrease in small pelagic fish catch in the eastern Indian Ocean during a negative IOD event and a decline in the catch rate of yellowfin tuna (YFT) in the western Indian Ocean during the positive IOD event^21^. These findings highlight the considerable influence of climatic oscillation on Indian Ocean fishery.

Given these considerations, this study explored the relationships of climatic oscillations (and their lagged versions) in the Indian Ocean with the rates of marlin catches by Taiwanese longliners. Because of high demand, tuna and tuna-like species command high commercial prices. Fisheries of such species contribute substantially to the global seafood industry and provide millions of people with employment and income. Tuna catches are often exported to satisfy consumer demand for products such as refrigerated tuna, canned tuna, and tuna sashimi. Consequently, research on tuna and tuna-like species has been conducted in all the oceans^22–24^. However, Information on how climatic oscillations and ocean conditions affect the catch rates and distribution of marlin species in the Indian Ocean remains limited. Marlin species, members of the Istiophoridae family, hold substantial ecological importance and fulfill several roles in marine ecosystems^25^. As apex predators, marlins shape the structure and dynamics of the food web by regulating the abundance and behavior of their prey. Beyond their ecological value^26^, marlins are primarily targeted for their flesh and fins, reflecting their commercial importance. Notably, the consolidation of two distinct species into a single species in the Indo–Pacific and Atlantic Oceans was discovered through genetic divergence analyses^42^ corroborated by the outcomes of tagging experiments, which have provided evidence of the migratory behavior of the blue marlin across the Pacific Ocean^43^. Given the ecological and economic value, understanding how climatic oscillations affect marlin fishery is crucial. Thus, this study investigated the influence of climatic oscillations (and their lagged versions) in the Indian Ocean on the catch rate of different marlin species. We hypothesized that climatic oscillations influence the catch rates of marlin species in the Indian Ocean after various time lags.

## Results

### Variability in yearly catch rate

The catch rates for the striped, blue, and silver marlin fluctuated significantly between 1994 and 2016. The average catch rates for these species were 1.73, 2.87, and 0.21 individuals per hook, respectively (Supplementary Fig. S1). The striped marlin catch rate exceeded the average in 1994, 1996, 2001–2005, 2012–2013, and 2016 and peaked in 2004 at 4.18 individuals per hook. The blue marlin catch rate exceeded the average in 2002–2006, 2012–2013, and 2015–2016 and peaked in 2012 at 6.22 individuals per hook. The silver marlin catch rate hovered near the average during 1999–2008, except in 2002, when it fell to 0.27 individuals per hook. The silver marlin catch rate peaked in 2007 at 0.72 individuals per hook.

### Relations between catch rate and climatic oscillations

Various marlin catch rates were correlated with climatic indices after different lag periods. For the striped marlin, substantial correlations were observed with the IOD after 0-, 5-, 7-, and 8-year lags (Table 1). The strongest negative correlation (r =  − 0.416) was that with the PDO after a 3-year lag, whereas the strongest positive correlation (r = 0.392) was that with the MJO at 0 year lag. Additionally, the SIOD exhibited significant correlations after lags of 0, 2, 3, 4, 5, 6, and 8 years, with the strongest negative (r =  − 0.291) and positive (r = 0.318) correlations with catch rate oscillation after 3- and 6-year lags, respectively.

**Table 1:** Correlations between the catch rates of three marlin species and different climatic oscillations and their lags

Lag	Striped Marlin				Blue Marlin				Silver Marlin			
	IOD	PDO	MJO	SIOD	IOD	PDO	MJO	SIOD	IOD	PDO	MJO	SIOD
0	**− 0.168**	**− **0.013	**0.392 ~ **	**− 0.158**	**− **0.068	**− **0.055	**0.251**	0.068	**− **0.031	**− 0.148**	0.093	0.633**
**− **1	0.025	**0.111**	**− **0.074	**− **0.073	**− **0.014	**− 0.139**	**− 0.147**	0.067	**− 0.174**	**0.158**	**0.167**	0.528**
**− **2	**− **0.031	0.058	0.044	**− 0.265**	0.081	**− 0.292**	0.031	**− 0.185**	**− 0.221**	**0.243**	**0.141**	0.047
**− **3	**− **0.079	− 0.416**	**− **0.086	**− 0.291**	0.439**	− 0.497*	0.034	**− 0.231**	**− 0.192**	**0.143**	**0.211**	**− 0.129**
**− **4	0.017	**0.346**	**− 0.218**	**− 0.111**	**0.248**	− 0.581**	**− 0.114**	**− **0.021	**− **0.046	**0.155**	0.007	**− 0.343**
**− **5	**0.181**	**− 0.391 ~ **	**− 0.152**	**0.276**	**0.353 ~ **	− 0.467*	− 0.265*	**0.308**	0.056	**− **0.096	0.342**	− 0.534**
**− **6	0.089	0.006	**0.158**	0.318**	**0.325**	**− 0.242**	0.046	0.319**	0.061	**− **0.096	**− **0.066	**− 0.254**
**− **7	**0.205**	**0.373 ~ **	0.052	0.016	**0.173**	**− 0.139**	**− 0.107**	0.006	**0.256**	**− **0.041	**− 0.205**	**− 0.231**
**− **8	**− 0.188**	**0.236**	**0.132**	**− 0.133**	**0.151**	**− 0.123**	**0.128**	0.081	0.078	**− 0.177**	**− **0.001	**− 0.115**

Significance level: *** 0.001, ** 0.01, * 0.05, ~ 0.1

Selected correlations are highlighted in bold.

The blue marlin was significantly correlated with the IOD after 3-, 4-, 5-, 6-, 7-, and 8-year lags, with the strongest positive correlation (r = 0.439) being that with the 3-year lag. The PDO was significantly correlated with blue marlin catch after 1-, 2-, 3-, 4-, 5-, 6-, 7-, and 8-year lags, with the strongest negative correlation (r =  − 0.581) after a 4-year lag. Significant correlations with the MJO were observed after 0-, 1-, 4-, 5-, 7-, and 8-year lags, with the strongest positive correlation (r = 0.251) after a 0-year lag. The SIOD showed significant correlations at lags of 2, 3, 5, and 6 years. The strongest negative correlation (r =  − 0.231) was found after a 4-year lag, while the strongest positive correlation (r = 0.319) was found after a 6-year lag.

The silver marlin catch rate displayed significant correlations with the IOD after 1-, 2-, 3-, and 7-year lags, with the strongest negative correlation (r =  − 0.221) being that after the 2-year lag. The PDO exhibited significant correlations after 0-, 1-, 2-, 3-, 4-, and 8-year lags, with the strongest positive correlation (r = 0.243) being that with the 2-year lag. The MJO exhibited significant correlations with silver marlin catch rate after 1-, 2-, 3-, 5-, and 8-year lags, with the strongest positive correlation (r = 0.342) being that with the 5-year lag. The SIOD displayed significant correlations after 0-, 1-, 3-, 4-, 5-, 6-, 7-, and 8-year lags, with the strongest positive correlation (r = 0.633) being at 0-year lag.

### Influence of selected climatic oscillations on catch rate

Table 2 presents the climatic oscillations that explained the highest deviance in the catch rates of the three marlin species, as determined by generalized additive model (GAM) analysis (Supplementary Table S1). For the striped marlin, the most influential factors were the IOD after a 7-year lag, the PDO after a 3-year lag, the SIOD after a 6-year lag, and the MJO after a 0-year lag. These factors respectively explained 18.6%, 60.7%, 38.7%, and 40.5% of the deviance in striped marlin catch rates. The blue marlin catch rates were primarily explained by the IOD at a 3-year lag, the PDO at a 4-year lag, the SIOD at a 6-year lag, and the MJO at a 5-year lag, accounting for 37.7%, 44.6%, 30%, and 34.7% of the deviance, respectively. For the silver marlin, the most substantial contributors were the IOD at a 7-year lag, the PDO at a 2-year lag, the SIOD at a 0-year lag, and the MJO at a 5-year lag, explaining 37.1%, 54.9%, 77.3%, and 60.1% of the deviance, respectively.

**Table 2:** Lags of climatic oscillations with the greatest contribution to the deviance in catch rate, as determined from GAM analysis.

Species	IOD	PDO	SIOD	MJO
Striped marlin	**− **7	**− **3	**− **6	0
Blue marlin	**− **3	**− **4	**− **6	**− **5
Silver marlin	**− **7	**− **2	0	**− **5

### Relationships between catch rates and selected climatic oscillation variability

**Figure 1:**
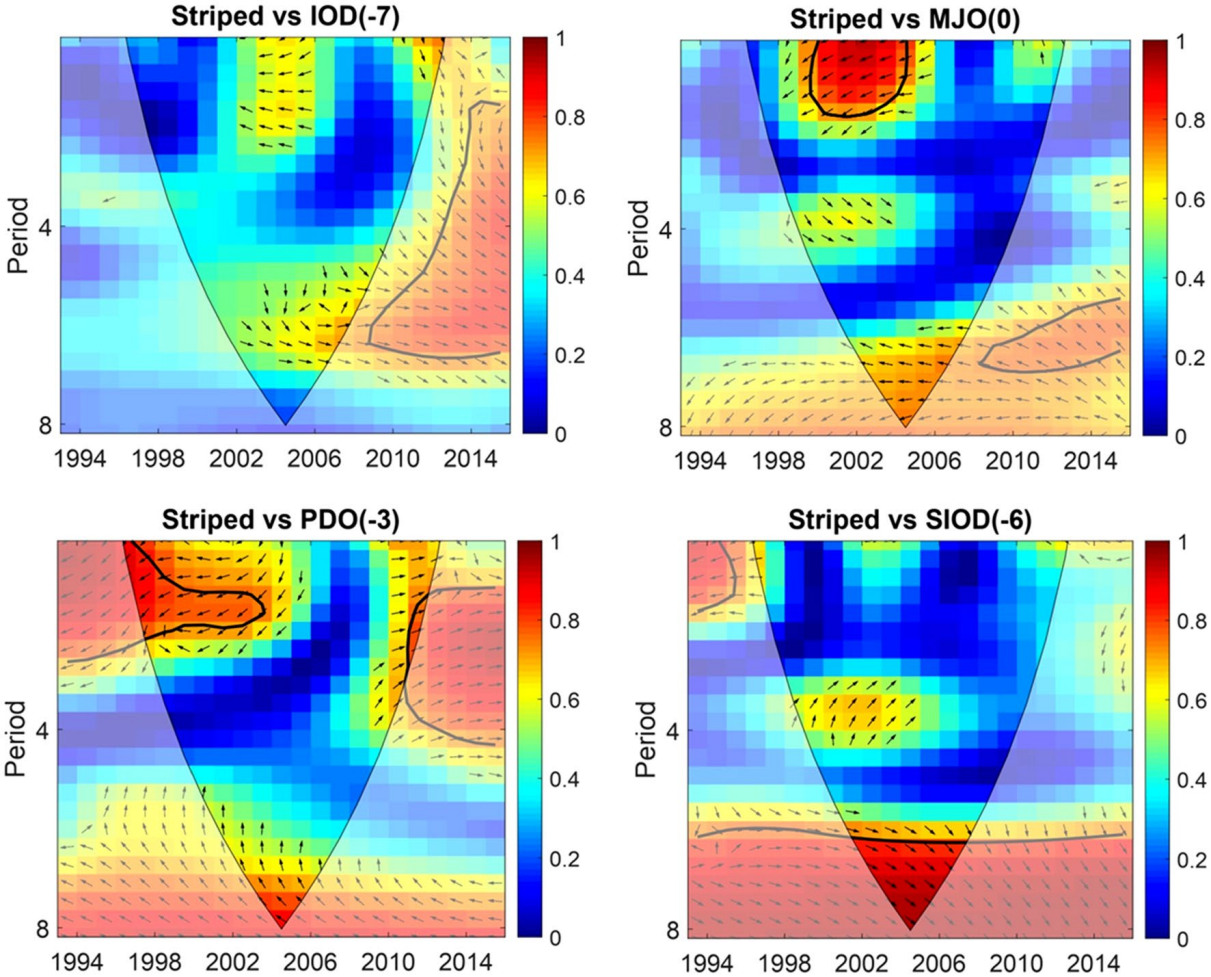
Inter-relation between striped marlin catch rate & selected climatic oscillation (from GAM) variability. Time is indicated as 1994–2016 in the y-axis. 0 (Lowest—Blue) to 1 (Highest—Red) in the legend indicates the degree of inter-relation.

Two phases of strong interrelations were observed between the striped marlin catch rate and the PDO at a 3-year lag: one from 1998 to 2005 (negative interrelation) and another from 2010 to 2012 (positive interrelation). A phase of strong interrelation was observed with the MJO after a 0-year lag, from 1999 to 2005 (negative interrelation). Additionally, one phase of strong interrelation was observed with the SIOD at a 6-year lag, from 2001 to 2007 (positive interrelation; Fig. 1).

**Figure 2:**
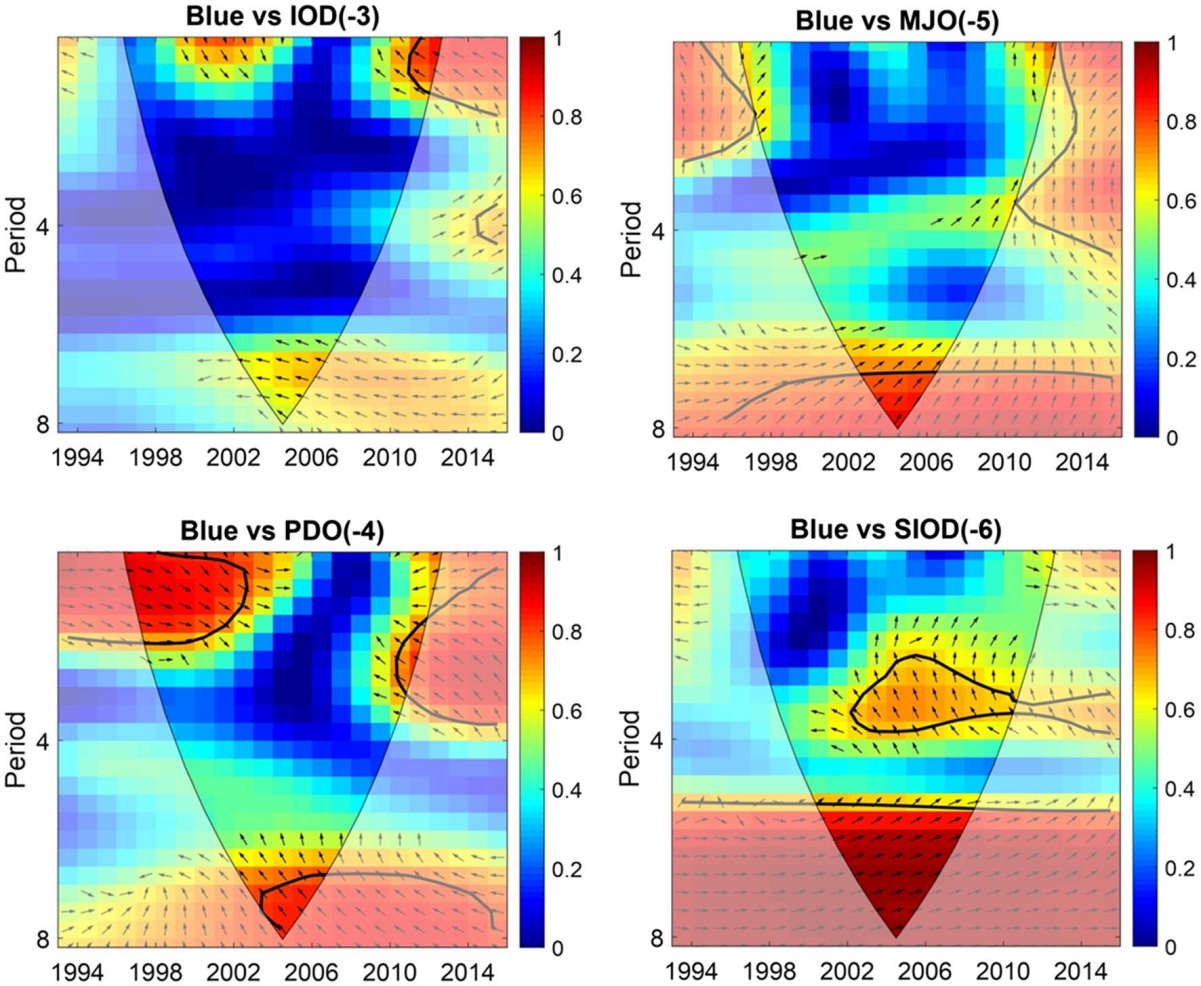
Inter-relation between blue marlin catch rate & selected climatic oscillation (from GAM) variability. Time is indicated as 1994–2016 in the y-axis. 0 (Lowest—Blue) to 1 (Highest—Red) in the legend indicates the degree of inter-relation.

A strong interrelation between blue marlin catch rate and the IOD at a 3-year lag occurred between 2010 and 2012 (positive interrelation). Two phases of strong interrelations with the PDO at a 4-year lag were observed, occurring between 1998 and 2002 (positive interrelation) and between 2002 and 2010 (negative interrelation). The MJO at a 5-year lag exhibited a single phase of strong interrelation with blue marlin catch rates, from 2001 to 2008 (positive interrelation). Finally, the SIOD at a 6-year lag exhibited two phases of strong interrelation: the first between 2001 and 2004 (3–4 years, positive interrelation) and the second between 2005 and 2010 (5–7 years, positive interrelation; Fig. 2).

**Figure 3:**
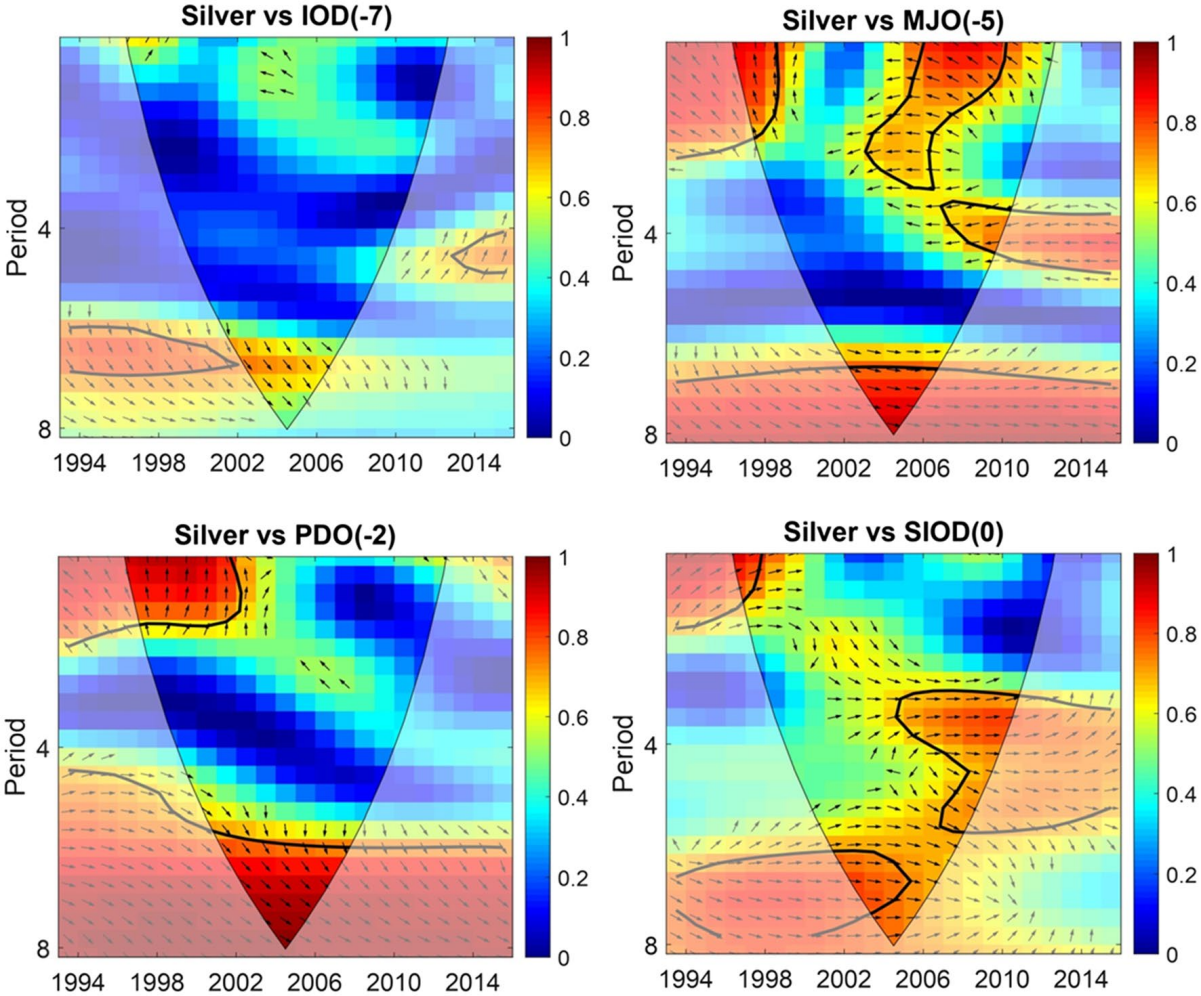
Inter-relation between silver marlin catch rate & selected climatic oscillation (from GAM) variability. Time is indicated as 1994–2016 in the y-axis. 0 (Lowest—Blue) to 1 (Highest—Red) in the legend indicates the degree of inter-relation.

Two phases of strong relations were observed between silver marlin catch rates and the PDO at a 2-year lag from 1998 to 2007 (positive interrelation). The MJO at a 5-year lag displayed three phases of strong interrelations with silver marlin catch rates from 1998 to 2000 (positive interrelation) and 2003–2010 (negative interrelation). The SIOD at a 0-year lag demonstrated three phases of positive interrelation with silver marlin catch rates, namely, 1997–1999, 2000–2006, and 2004–2008 (Fig. 3). These strong relations (wavelet coherence value) ranged from 0.7 to 1.

### Combined effects of climatic oscillation on catch rates

Examination of the combined effects of selected climatic oscillations with various time lags revealed no collinear effects in GAM analysis (Supplementary Fig. S2). The combination of the PDO with a 3-year lag and MJO with a 0-year lag explained the largest proportion of the deviance (72.1%), with an adjusted R^2^ of 0.61 among all the other climatic oscillations (Table 3, Supplementary Table S2). For the blue marlin, the combination of the IOD with a 3-year lag and MJO with a 5-year lag explained the largest proportion of the deviance (60.3%), with an adjusted R^2^ of 0.5. For the silver marlin, the combination of the PDO with a 2-year lag and IOD with a 7-year lag explained the largest proportion of the deviance (83.2%), with an adjusted R^2^ of 0.71. QQ-plots for the selected models for each species are displayed in Supplementary Fig. S3.

**Table 3:** Performance of models based on the largest contributing oscillation variables to the catch rate of each marlin species.

Species	Model	Adjusted R^2^	Deviance (%)	*P*(f)
Striped marlin	PDO(**− **3), MJO(0)	0.61	72.1	< 0.001
Blue marlin	IOD(**− **3), MJO(**− **5)	0.52	60.3	
Silver marlin	IOD(**− **7), PDO(**− **2)	0.71	83.2	

### Variability in catch rates across phases of climatic oscillations

Table 4 presents the phase-wise changes in the catch rates of the three marlin species with selected climatic oscillations. During the positive and negative phases of the IOD with a 7-year lag, the catch rate for the striped marlin increased by 21.8% and decreased by 11.6%, respectively. The blue marlin catch rate exhibited similar trends during the positive and negative phases of the IOD with a 3-year lag, increasing by 2.9% and decreasing by 1.8%, respectively. However, the silver marlin catch rate exhibited an opposite trend; during the positive and negative phases of the IOD with a 7-year lag, the catch rate decreased by 5.9% and increased by 3.1%, respectively. Striped marlin catch rates during the positive and negative phases of the PDO with a 3-year lag decreased by 21.5% and increased by 7.6%, respectively. Blue marlin catch rates displayed similar trends during the positive and negative phases of the PDO with a 4-year lag, where the catch rate decreased by 28.2% and increased by 9.9%, respectively. However, silver marlin catch rates during the positive and negative phases of a 2-year lagged PDO decreased by 0.6% and increased by 0.2%, respectively. Striped marlin catch rates during the positive and negative phases of a 6-year lagged SIOD decreased by 1.1% and increased by 1.5%, respectively. A similar pattern was observed for the blue marlin during the positive and negative phases of a 6-year lagged SIOD where the catch rate decreased by 1.2% and increased by 1.8%, respectively. Conversely, the silver marlin catch rates during the positive and negative phases of a 0-year lagged SIOD increased by 38.1% and decreased by 29.3%, respectively. Striped marlin catch rates during the positive and negative phases of the MJO (0 lag) decreased by 3.1% and increased by 10.3%, respectively. The blue marlin demonstrated similar trends during the positive and negative phases of a 5-year lagged MJO where catch rates decreased by 16.1% and increased by 5.5%, respectively. However, a different pattern was observed for the silver marlin during the positive and negative phases of a 5-year lagged MJO where the catch rates increased by 5.01% and decreased by 0.4%, respectively.

**Table 4:** Phase-wise catch rate changes of three marlin species for climatic oscillations with various lags.

Striped marlin		Blue marlin		Silver marlin	
Oscillations phases	Changes (%)	Oscillations phases	Changes (%)	Oscillations phases	Changes (%)
+ IOD (− 7)	+ 21.8	+ IOD (− 3)	+ 2.9	+ IOD (− 7)	− 5.9
− IOD (− 7)	− 11.6	− IOD (− 3)	− 1.8	− IOD (− 7)	+ 3.1
+ PDO (− 3)	− 21.5	+ PDO (− 4)	− 28.2	+ PDO (− 2)	− 0.6
− PDO (− 3)	+ 7.6	− PDO (− 4)	+ 9.9	− PDO (− 2)	+ 0.2
+ SIOD (− 6)	− 1.1	+ SIOD (− 6)	− 1.2	+ SIOD (0)	+ 38.1
− SIOD (− 6)	+ 1.5	− SIOD (− 6)	+ 1.8	− SIOD (0)	− 29.3
+ MJO (0)	− 3.1	+ MJO (5)	− 16.1	+ MJO (5)	+ 5.01
− MJO (0)	+ 10.3	− MJO (5)	+ 5.5	− MJO (5)	− 0.4

## Discussion

Climatic oscillations and their associated anomalies exert a profound influence on marine ecosystems, particularly affecting the migration and population dynamics of apex predators such as tuna and marlins. The observed phenomena caused by the IOD in the Indian Ocean are influenced by recurrent interannual variability in SST. This variation in SST induces anomalies in wind patterns and precipitation levels. Connections with the El Niño phenomenon contribute to an overall increase in the Indian Ocean’s temperature. This warming is driven by shifts in cloud cover and wind patterns, which result from changes in the ascending and descending sections of the Walker circulation^27^. Such climatic phenomena affect multiple facets of the ecology of the Indian Ocean, including ecosystem function, fishery resources, and carbon sequestration^28^. For example, IOD events have been linked to changes in the catch rates of YFT in the Indian Ocean^21^. The findings of this study align with observations of climatic oscillations being associated with fluctuations in the catches of these three marlin species in the Indian Ocean. The catch rates of all three marlin species were associated with the lags of oscillations, displaying a periodic pattern.

This study demonstrated the crucial influence of the lagged effects of climatic oscillations on the catch rates of these three marlin species, revealing a delay between these oscillations and their effects on fish populations. The majority of the time-lagged effects of four climatic oscillations exhibited a positive or negative correlation with the catch rates of marlin species. The time-lagged effects of ocean conditions or climatic oscillations can be attributed to several factors.

For instance, to incremental changes in ocean conditions, fish populations can either acclimate or adapt. Acclimation refers to short-term adjustments made by individuals, whereas adaptation refers to long-term genetic changes within populations. Both processes can be time-consuming and result in lagged distribution shifts^29^. Furthermore, alterations in ocean conditions—such as vegetation or water column depth or structure—can directly affect the availability of suitable habitats for fish. Fish may require time to discover and become acclimated to new habitats, resulting in a lag in their distributional response^30^. Our findings on the time-lagged effect on catch rates are corroborated by a study on Pacific billfish^31^. Another study demonstrated that lagging the effect of the North Atlantic oscillation (NAO) by 1 year increased the ability of the NAO to explain the distribution of North Atlantic billfish from 13 to 33%^32^. The effects of climatic oscillations for long-lived species such as marlins are expected to have long lags. For small pelagic fishes with limited lifespans, such as sardines and anchovies, the lag can be minimal. Teixeira et al.^33^ found a lag of less than a year in the effect of the NAO in the preceding winter on the recruitment of the European sardine. Conversely, Faillettaz et al.^34^ observed the effect of a lag of 16 years of the Atlantic Multidecadal Oscillation (AMO) on the recruitment for the long-lived bluefin tuna. Baez et al.^35^ found that the distribution of YFT in the Indian Ocean is substantially affected by the lagged effects of climatic oscillations. They found that the IOD, PDO, and MJO were most strongly correlated with the YFT catch rate in the Indian Ocean after 6, 5, and 5 years, respectively. These slight variations in the lag in the effect on YFT highlight the sensitivity of different fish species to changes in ocean conditions. Some species may respond quickly to environmental changes^36,37^. The aforementioned studies support our findings, which suggest the importance of lagged effects on marlin catch rates in the Indian Ocean. Our results demonstrate that marlin catch rates or distributions may not change immediately after the onset of a climatic oscillation but do change after a delay as fish adjust their distribution in response to altered ocean conditions^38^.

In the tropical Indian Ocean, a negative IOD event can lead to a reduction in the depth of the thermocline, concentrating productivity at the sea surface. The thermocline-SST feedback has a negative impact on the occurrence and intensity of positive Indian Ocean Dipole (IOD) events, but it is favorable for negative IOD events^39^. Strong IOD events primarily stem from the coupling between the thermocline and SST, fostering a highly interactive relationship with the atmosphere. Conversely, weak IOD events are merely a response to surface winds, lacking the dynamic coupling observed of their strong counterparts^40^. The striped marlin catch rate was associated with the IOD across the Indian Ocean, whereas no significant associations were observed with the catch rates of the blue or silver marlin. These three species exhibit distinct depth distributions and vertical movement patterns. Specifically, blue marlins inhabit deeper waters (approximately 1000 m) than do striped (approximately 200 m) and silver (approximately 900 m) marlins. Given these differences, during an IOD event, striped marlin populations residing in shallower depths might be more susceptible to environmental fluctuations. This susceptibility could lead to higher catch variability during positive IOD phases, as observed in this study. Thus, species-specific differences were also observed in this study.

Climate variability may similarly affect multiple species. Lynam et al.^41^ examined the associations between the population levels of three jellyfish species—*Aurelia aurita*, *Cyanea lamarckii*, and *Cyanea capillata*—and the NAO in the North Sea. They found a significant negative relationship between the abundance of *A. aurita* and *C. lamarckii* and the NAO near northwestern Denmark and eastern Scotland. This association might be due to hydroclimatic fluctuations arising from atmospheric influences on wind stress, temperature, and currents. However, the effect on *C. capillata* was nonsignificant. Rubio et al.^42^ examined the effect of the NAO on albacore and YFT fishing yields in the northeastern Atlantic Ocean, finding significant positive associations between the NAO and catch rates for both species. However, IOD-induced changes in the marine environment predominantly appear in the equatorial area and may have less of an effect on the blue and silver marlin populations, which are situated farther from the equator. The relation between the PDO and the Indian Ocean seems to have influenced the striped marlin catch, underscoring the interconnectedness of global climate patterns and marine ecosystems. In addition, tuna fishery in the Indian Ocean may experience a noticeable decrease if it is not synchronized with the PDO^35^. Large pelagic fish like billfish, tuna, and shark species experience substantial environmental fluctuations throughout their life cycle due to their large-scale migration patterns. In order to effectively understand and forecast the long-term abundance variations of these species, it is plausible to consider employing large-scale climate indices that integrate multiple physical variables as a suitable proxy. Given the enhanced comprehension of the teleconnection pattern between the oceans and atmosphere within the context of global climate change, it is imperative to emphasize the trans-basin impact of climate patterns on the forecasting of large-scale migratory species, akin to the basin climatic oscillations, in forthcoming times.

The blue marlin, known for its migratory behavior, dwells in various tropical, subtropical, and temperate waters from 45° N to 45° S^43^. Therefore, the PDO could substantially affect this species, leading to variations in catch rates in the Indian Ocean. Baez et al.^35^ indicated a lagged effect on YFT catches in the Indian Ocean of the interaction between the PDO and SIOD. This lagged effect may be associated with favorable recruitment, enhanced larval survival, or improved YFT spawning. Observations suggest that a negative phase of the PDO or a positive phase of the SIOD may increase fish stock abundance after 3 to 6 years. Conversely, a positive phase of the PDO or a negative phase of the SIOD may diminish stock abundance after 3 or 6 years. Time series data from another study^44^ on YFT in the Indian Ocean revealed a correlation between the standardized catch per unit effort, distribution, and the influence of the PDO in the Indo–Pacific Ocean, indicating the significance of trans-oceanic teleconnections. The effect on blue marlins could be similar because both YFT and blue marlins are large pelagic predatory fishes with extensive migration routes. Studies have documented the influence of the PDO on global SST and on the recruitment and abundance of fishes^45^. Therefore, the PDO also substantially affects MLD and net primary productivity from the Pacific Ocean to the Indian Ocean. This influence further affects the intensity of the monsoon system^46^. During positive PDO events, the temperatures in the subsurface layer of the Indian Ocean, specifically at depths ranging from 100 to 320 m, tend to be lower. Similarly, the thermocline depths in the same region tend to be greater during these events. These changes may explain the increased blue marlin catch in the Indian Ocean during such events.

Studies have underscored the role of the SIOD, another mode of SST variability in the subtropical Indian Ocean region, in initiating the tropical IOD^47^. Our understanding of the specific effects of the SIOD on fish catches remains less comprehensive as that of the effects of other climatic patterns such as the IOD or ENSO. Our results reveal that the variability in silver marlin catch is strongly affected by both the positive and negative phases of the SIOD. One study highlighted a cyclical feedback mechanism between the IOD and SIOD, particularly in tropical and subtropical regions and when ENSO influences are weak or absent. In this feedback cycle, the presence of positive (negative) SIOD tends to promote a positive (negative) IOD, whereas a positive (negative) IOD tends to promote a negative (positive) SIOD^48^. This cycle strongly supports our results for the silver marlin, whose peak catch variability (38.1%) was caused by a positive SIOD and occurred during a positive IOD event (typically associated with reduced catches). This study demonstrated that highly mobile fishes, such as marlin species, are susceptible to the effects of climatic oscillations.

The findings of this study on the relationship between marlin catch and climatic oscillations provide crucial insights for the management of marlin species, particularly through the use of early warning systems (EWSs)^9^. Extreme climatic events can have substantial effects on marine ecosystems and fishery stock abundance, highlighting the necessity of an EWS. By identifying relationships between climatic oscillations and marlin catch rates, this study provides potential indicators of shifts in fishery stock and ecosystems. This valuable information can aid governments in planning and implementing strategies to mitigate adverse effects on fishing industries and coastal communities, thereby promoting the resilience of marine ecosystems and enhancing socioeconomic welfare.

## Conclusion and remarks

This study examined the effects of climatic oscillations on the catch rates of the striped, blue, and silver marlin in the Indian Ocean. The catch rate variability of the silver marlin differed notably from that of the striped and blue marlin. Our results indicate the role of climatic oscillations and their lagged effects on marlin catch rates, supporting our hypothesis. Our findings suggest that marine scientists, policymakers, and coastal communities should acknowledge the effects of climatic oscillations on ocean conditions. Such an understanding could improve the management of marine resources, forecasting of changes in ocean ecosystems, and development of strategies to adapt to and mitigate the effects of climate variability on the oceans. Such effects can be far-reaching on marine biodiversity, coastal economies, and the overall health of the climate system.

## Methods

### Data collection

#### Marlin fishery data

**Figure 4:**
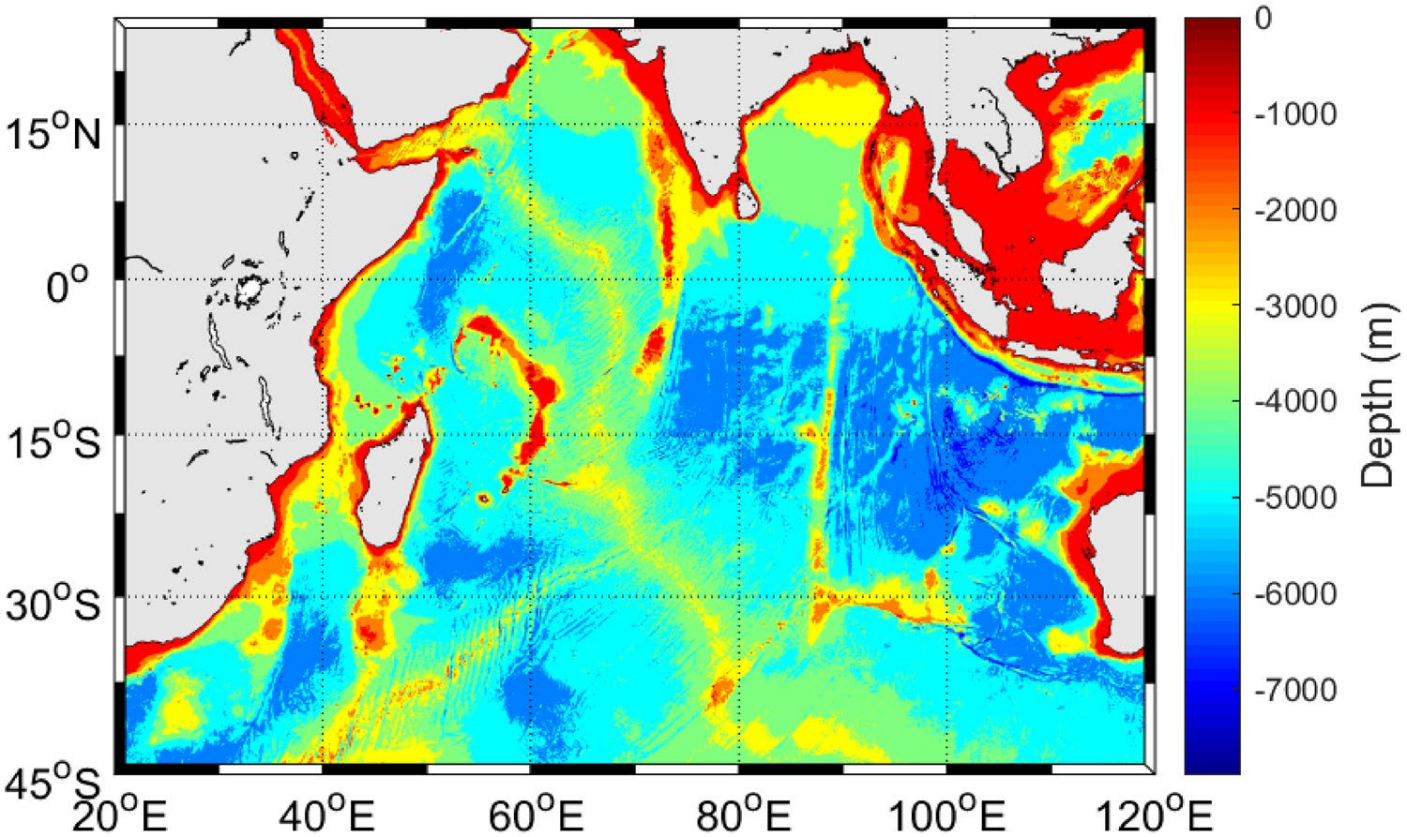
Study area.

Monthly fishery data for the striped, blue, and silver marlin were collected from the logbooks of Taiwanese large-scale long-line fishing vessels (deep-water fishing vessels with volumes > 100 gross registered tonnage and lengths > 24 m); these logbooks were obtained from the Overseas Fisheries Development Council for the period 1994–2016. The spatial coverage of the data was 25° N–44° S and 20° E to 120° E at a resolution of 1° × 1° (Fig. 4). The logbooks data included the year, month, latitude, longitude, and number of catches and the number of hooks used. However, data related to hook depth and operation time were not available. Small-scale vessels (those fishing primarily in offshore waters, with volumes < 100 gross registered tonnage and lengths < 24 m) were not included in this study due to the unavailability of data for the specified period.

#### Climatic oscillation data

Data on four climatic oscillations—the IOD, SIOD, MJO, and PDO—were collected for the period 1994–2016. For each climatic oscillation, lags of up to 8 years were also considered. The sources of the climatic oscillation data are provided in Supplementary Table S3.

### Data analysis

#### Yearly catch rate variability

The catch rate of each marlin species in this study was calculated using the following formula^49^:

1 $$\mathrm{Catch rate}=\frac{\mathrm{Catch in number }({\text{Catch}})}{\mathrm{Number of hooks deployed }\left({\text{Effort}}\right)}$$

The study used autoregressive integrated moving average time series analysis (ARIMA)^50^ to assess yearly catch rate variability. ARIMA combines autoregressive (AR) and moving average (MA) components, along with differencing, to capture the inherent patterns within the time series under investigation^50^. This analysis was performed in the R environment (version 3.6.0) using the “ts” function of the “tseries” package^51^ and the “cpt.meanvar” function of the “changepoint” package^52^. Henceforth, this catch rate value was used for the forthcoming analysis.

#### Relations between catch rate and climatic oscillations

The Pearson correlations between the catch rates of each species and the climatic oscillations (including their lags) were analyzed^53^. This analysis was performed in the R environment (version 3.6.0) using the “cor.test” function of the “corrr” package. Only climatic oscillations with an absolute correlation value of 0.1 or more with the catch rate of any marlin species were selected for further analysis^35^. The Pearson correlation coefficients were calculated as follows:

2 $$ {\text{r}} = \frac{{\mathop \sum \nolimits_{{{\text{i}} = 1}}^{{\text{n}}} \left( {{\text{X}}_{{\text{i}}}  - {\text{X}}} \right){\text{(Y}}_{{\text{i}}}  - {\text{Y)}}}}{{\sqrt {\mathop \sum \nolimits_{{{\text{i}} = 1}}^{{\text{n}}} \left( {{\text{X}}_{{\text{i}}}  - {\text{X}}} \right)^{2} } \sqrt {\mathop \sum \nolimits_{{{\text{i}} = 1}}^{{\text{n}}} \left( {{\text{Y}}_{{\text{i}}}  - {\text{Y}}} \right)^{2} } }} $$

In this equation, n is the sample size, X_i_ and Y_i_ are individual sample points indexed by i, and X and Y are sample mean values.

A higher correlation coefficient indicates the presence of collinearity between pairwise variables. A Pearson correlation coefficient close to 0.8 suggests the presence of collinearity^54^. Climatic oscillations exhibiting an absolute correlation value greater than 0.1 were selected as potential explanatory variables with non-linear impacts on marlins. Furthermore, only the time delays of the selected climatic oscillations that had an absolute correlation value greater than 0.1 were chosen for the subsequent analysis. The correlation coefficient ranges from − 1 to + 1. The magnitude of a correlation indicates the direction of changes in one variable resulting from changes in another^55^. This test has the potential to reveal the significant association between climatic variables and fish catch at different lag periods, suggesting that the distribution and abundance of fish, including catch, are reliant on ecosystem characteristics^56^. Examining the correlation structure of a time series can unveil inherent patterns such as seasonality or trends^57^.

#### Effect of selected climatic oscillations on catch rate

The effects of selected climatic oscillations on the striped, blue, and silver marlin catch rates were analyzed using the GAM^58^. GAM allows one to analyse non-linear relationships and capture complex patterns in the data, utilising smooth functions to account for potential non-linearities in predictor variables. GAM results are associated with “deviance explained,” a metric quantifying the proportion of variability in the dependent variable accounted for by the model, signifying the reduction in deviance compared to a null model; higher explained deviance values denote a stronger ability of the GAM to explain observed variability in the dependent variable^58^. For each species, one GAM was constructed for each climatic oscillation, with the climatic oscillation serving as the predictor variable and catch rate serving as the response variable^59^. This analysis was performed in the R environment (version 3.6.0) using the “smoothing” function of the “mgcv” package^59^. The weightage of climatic oscillation variables was ranked on the basis of deviance explained and the Akaike information criterion (AIC). AIC is a metric used to compare model results by quantifying the trade-off between model fit and complexity, with lower AIC values indicating a better fit and parsimonious model complexity^58,59^. Only the models with the greatest deviance explained and lowest AIC were selected for further analysis. Each GAM was constructed using the following formula:

3 $${\text{GAM}}:\left(\mathrm{Catch rate}+{\text{c}}\right) \sim \mathrm{ s}(\mathrm{Predictor variable})$$

where c is a constant value of 0.1 and s is the smoothing function. The climatic oscillation with the greatest effect on the catch of each species was considered the chosen predictor for the final analysis.

#### Interrelation between catch rate and selected climatic oscillation variability

The interrelation between the yearly catch rate variability of each marlin species and the yearly variability of their respective selected climatic oscillations was analyzed using cross-wavelet time series analysis^60^. This analysis was performed in the R environment (version 3.6.0) using the “wtc” function of the “biwavelet” package^61^. Following Grinsted et al. (2004)^62^, the cross-wavelet coherence of two time series (i.e., yearly catch rate and climatic oscillation variability) was defined as follows:

4 $${R}_{n}^{2}\left(s\right)=\frac{{\left|S\left[{s}^{-1}{W}_{n}^{XY}\left(s\right)\right]\right|}^{2}}{S{\left({s}^{-1}{W}_{n}^{X}\left(s\right)\right)}^{2}\cdot {S\left[{s}^{-1}{W}_{n}^{Y}\left(S\right)\right]}^{2}}$$

where W is the wavelet transform of the time series and S is a smoothing operator used to calculate average values. X and Y are two time series i.e. catch rate and climatic oscillation.

#### Combined effect of climatic oscillations on catch rate

This study used the GAM methodology to assess the combined effects of climatic oscillations with various lags on the catch rate of each species. Models were constructed separately for each species with all possible pairs of the selected climatic oscillation variables from the previous GAM analysis. Before modelling, pairs were constructed, the collinearity of the effects of any selected climatic oscillations on catch rate was assessed through the calculation of the variance inflation factor (VIF) in the R environment (version 3.6.0) by using the “vif” function of the “car” package. Only climatic oscillations that did not exceed a threshold VIF value of 5^63^ were selected for model construction.

GAMs were separately constructed from pairs of the selected climatic oscillation variables after the VIF analysis for each species; these analyses were performed in the R environment (version 3.6.0) using the “smoothing” function from the “mgcv” package. Each paired GAM was constructed using the following formula:

5 $${\text{GAM}}:\left(\mathrm{Catch rate}+{\text{c}}\right) \sim \mathrm{ s}\left(\mathrm{Climatic oscilaltion }1\right)+ {\text{s}}(\mathrm{Climatic oscilaltion }2)$$

*Phase-wise catch rate variability.* This study assessed the relation of the phase-wise catch rate variability of the three marlin species with the climatic oscillations. Each selected climatic oscillation was divided into positive and negative phases. The average catch rate throughout the study period was calculated to serve as the base catch rate for identifying the positive and negative phases. The average catch rates during the positive and negative phases were then calculated. The increase or decrease in catch rate of each species (%) during the positive and negative phases were calculated using the following formulas:

6 $$\mathrm{Positive phase}:\left(\mathrm{Catch rate changes}\right)= \frac{{\mathrm{Catch rate}}_{\mathrm{Positive phase}}- {\mathrm{Catch rate}}_{{\text{Average}}}}{{\mathrm{Catch rate}}_{{\text{Average}}}}\mathrm{ x }100$$

7 $$\mathrm{Negative phase}:\left(\mathrm{Catch rate changes}\right)= \frac{{\mathrm{Catch rate}}_{\mathrm{Negative phase}}- {\mathrm{Catch rate}}_{{\text{Average}}}}{{\mathrm{Catch rate}}_{{\text{Average}}}}\mathrm{ x }100$$

### Supplementary Information

Supplementary Information.

## Data Availability

For access to the data used in this study, please contact the following author: Ming-An Lee (malee@mail.ntou.edu.tw).
