# Supplementary material for: Impact of climatic oscillations on marlin catch rates of Taiwanese long-line vessels in the Indian Ocean
Source: Sci Rep. 2023 Dec 17;13:22438. doi: 10.1038/s41598-023-49984-4 (PMC10725878; doi:10.1038/s41598-023-49984-4)
Supplement: Supplementary file 1 — Supplementary Information. [file 41598_2023_49984_MOESM1_ESM.docx]

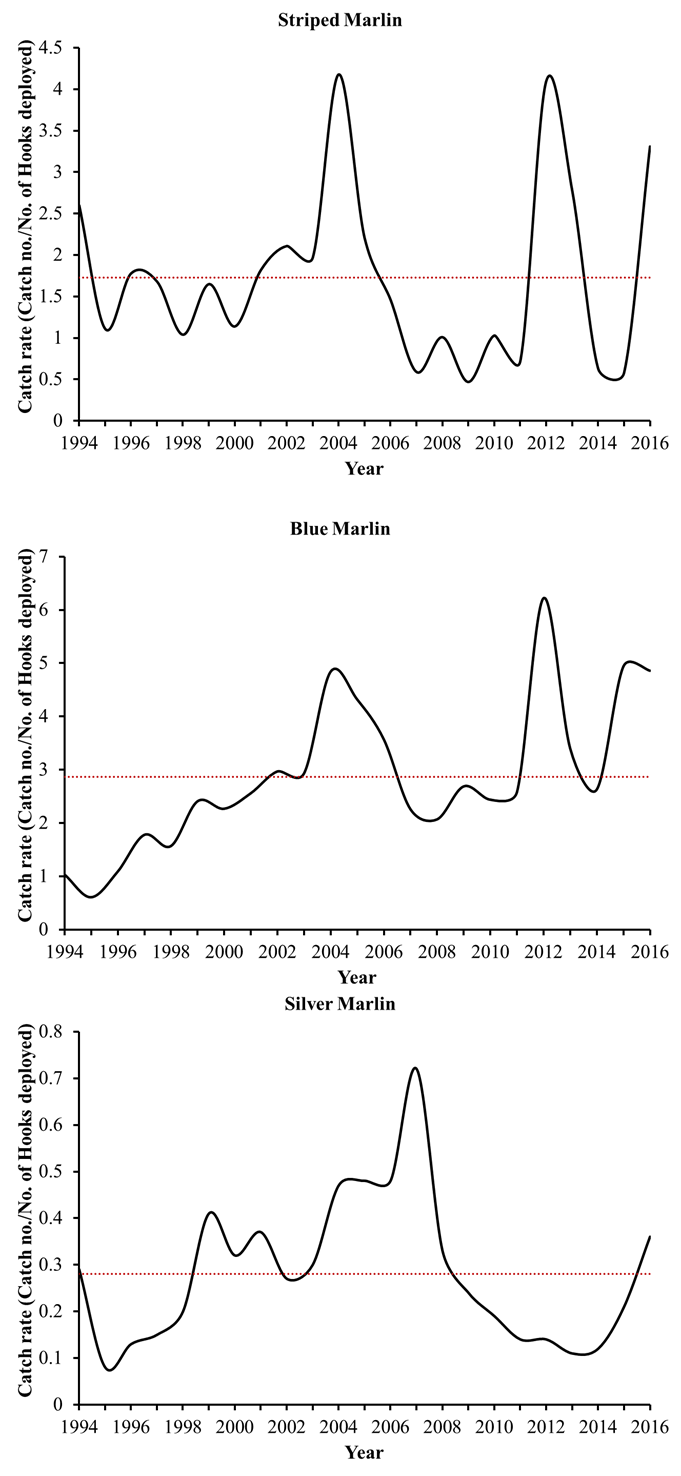


**Supplementary Fig. S1.** Yearly catch variability of three marlin species during the study period. The red dotted line indicates the average catch rate during 1994 to 2016.


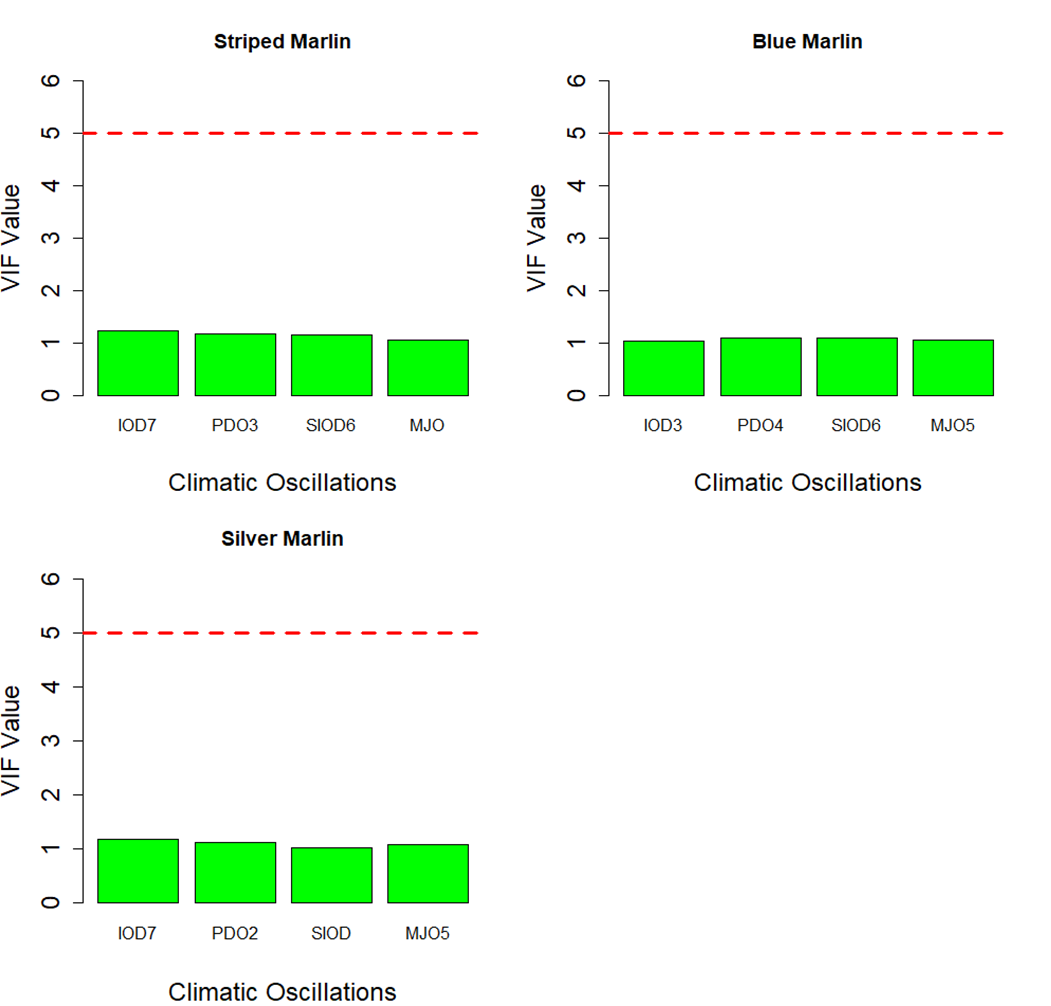


**Supplementary Figure S2.** VIF analysis to result from the collinearity test result for the selected climatic oscillations. Dotted red line indicates the threshold VIF value.


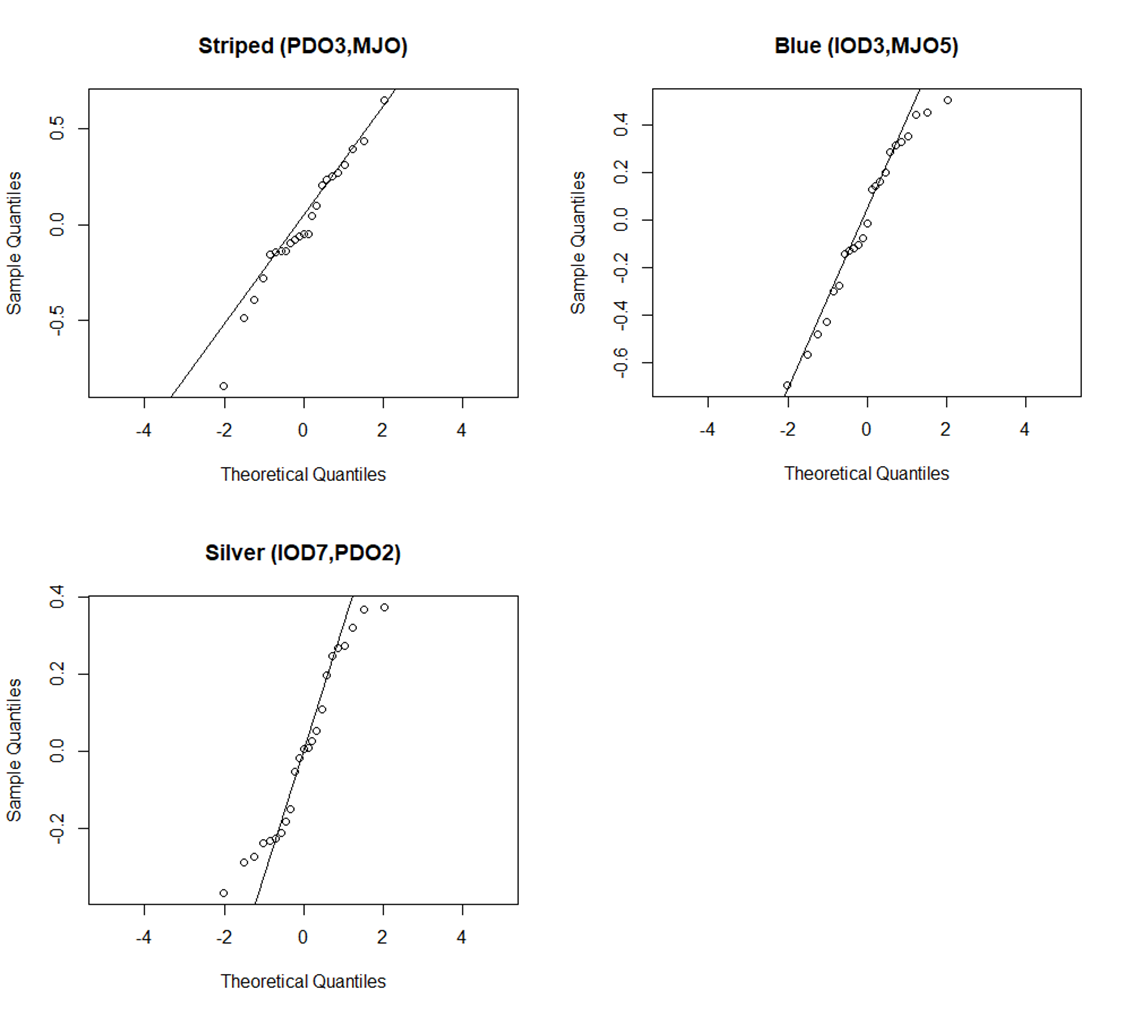


**Supplementary Figure S3.** QQ-plot for the selected model for each species from GAM analysis.

**Supplementary Table S1.** Assessment of the impact of climatic oscillations on the catch rate of three marlin species using GAM. Bolded ones are the selected ones.

| **Striped Marlin** | | | | | | | | |
| --- | --- | --- | --- | --- | --- | --- | --- | --- |
| **IOD** | | **PDO** | | **SIOD** | | **MJO** | | **Lag** |
| ***AIC*** | ***Dev*** | ***AIC*** | ***Dev*** | ***AIC*** | ***Dev*** | ***AIC*** | ***Dev*** |  |
| 72.6 | 2.8 |  |  | 72.7 | 2.5 | **67.0** | **40.5~** | 0 |
|  |  | 72.6 | 8.1 |  |  |  |  | -1 |
|  |  |  |  | 68.9 | 30.7 |  |  | -2 |
|  |  | **58.9** | **60.7*** | 71.3 | 8.4 |  |  | -3 |
|  |  | 69.2 | 24.0 | 73.1 | 1.2 | 72.2 | 4.8 | -4 |
| 72.5 | 3.2 | 61.9 | 58.7* | 71.5 | 7.5 | 72.6 | 5.8 | -5 |
|  |  |  |  | **66.5** | **38.7~** | 70.5 | 17.7 | -6 |
| **70.6** | **18.6** | 69.8 | 13.9~ |  |  |  |  | -7 |
| 72.3 | 7.8 | 72.1 | 5.6 | 72.9 | 1.7 | 72.9 | 1.8 | -8 |

**Significance level: ‘***’ 0.001, ‘**’ 0.01, ‘*’ 0.05, ‘~’ 0.1**

| **Blue Marlin** | | | | | | | | |
| --- | --- | --- | --- | --- | --- | --- | --- | --- |
| **IOD** | | **PDO** | | **SIOD** | | **MJO** | | **Lag** |
| ***AIC*** | ***Dev*** | ***AIC*** | ***Dev*** | ***AIC*** | ***Dev*** | ***AIC*** | ***Dev*** |  |
|  |  |  |  |  |  | 82.7 | 17.5 | 0 |
|  |  | 85.1 | 14.0 |  |  | 85.0 | 2.2 | -1 |
|  |  | 83.5 | 8.5 | 84.7 | 3.5 |  |  | -2 |
| **18.4** | **37.7** | 79.0 | 24.7* | 84.3 | 5.4 |  |  | -3 |
| 22.6 | 29.6 | **73.8** | **44.6**** |  |  | 85.2 | 1.3 | -4 |
| 24.0 | 22.9 | 79.3 | 27.1* | 83.2 | 9.5 | **78.9** | **34.7** | -5 |
| 22.6 | 21.4 | 84.1 | 5.9 | **80.6** | **30.0** |  |  | -6 |
| 23.7 | 12.0 | 85.1 | 2.0 |  |  | 85.3 | 1.2 | -7 |
| 25.2 | 0.1 | 85.2 | 1.5 |  |  | 85.2 | 1.6 | -8 |

**Significance level: ‘***’ 0.001, ‘**’ 0.01, ‘*’ 0.05, ‘~’ 0.1**

| **Silver Marlin** | | | | | | | | |
| --- | --- | --- | --- | --- | --- | --- | --- | --- |
| **IOD** | | **PDO** | | **SIOD** | | **MJO** | | **Lag** |
| ***AIC*** | ***Dev*** | ***AIC*** | ***Dev*** | ***AIC*** | ***Dev*** | ***AIC*** | ***Dev*** |  |
|  |  | -18.0 | 19.1 | **-36.8** | **77.3**** |  |  | 0 |
| -15.8 | 4.5 | -18.1 | 22.2 | -27.1 | 55.2* | -16.1 | 8.2 | -1 |
| -16.1 | 18.4 | **-25.1** | **54.9*** |  |  | -15.6 | 4.1 | -2 |
| -18.3 | 25.9 | -16.6 | 12.4 | -15.4 | 1.7 | -20.6 | 34.4 | -3 |
|  |  | -15.6 | 2.4 | -17.9 | 11.8 |  |  | -4 |
|  |  |  |  | -25.1 | 41.6 | **-26.2** | **60.1*** | -5 |
|  |  |  |  | -16.6 | 6.5 |  |  | -6 |
| **-19.0** | **37.1*** |  |  | -16.4 | 6.8 | -16.0 | 4.2 | -7 |
|  |  | -24.4 | 40.7* | -15.3 | 1.3 |  |  | -8 |

**Significance level: ‘***’ 0.001, ‘**’ 0.01, ‘*’ 0.05, ‘~’ 0.1**

**Supplementary Table S2.** Performance of the all possible paired models to explain the catch rate for all three marlins. Bolded ones are with the highest contributions.

| **Striped Marlin models** | **adjusted R2** | **Dev** |
| --- | --- | --- |
| IOD7,PDO3 | 0.41 | 56.3** |
| IOD7, SIOD6 | 0.23 | 38.1** |
| IOD7, MJO | 0.47 | 65.2** |
| PDO3, SIOD6 | 0.52 | 71.1** |
| **PDO3, MJO** | **0.61** | **72.1***** |
| SIOD6, MJO | 0.42 | 57.8** |

**Significance level: ‘***’ 0.001, ‘**’ 0.01, ‘*’ 0.05, ‘~’ 0.1**

| **Blue Marlin models** | **adjusted R2** | **Dev** |
| --- | --- | --- |
| IOD3, PDO4 | 0.36 | 46.9*** |
| IOD3, SIOD6 | 0.22 | 33.4*** |
| **IOD3, MJO5** | **0.5** | **60.3***** |
| PDO4, SIOD6 | 0.17 | 26.7*** |
| PDO4, MJO5 | 0.26 | 37.2*** |
| SIOD6, MJO5 | 0.22 | 34.1*** |

**Significance level: ‘***’ 0.001, ‘**’ 0.01, ‘*’ 0.05, ‘~’ 0.1**

| **Silver Marlin models** | **adjusted R2** | **Dev** |
| --- | --- | --- |
| **IOD7, PDO2** | **0.71** | **83.2***** |
| IOD7, SIOD22 | 0.26 | 44.2*** |
| IOD7, MJO5 | 0.43 | 61.2*** |
| PDO2, SIOD | 0.41 | 50.7*** |
| PDO2, MJO5 | 0.54 | 62.5*** |
| SIOD, MJO5 | 0.51 | 34.1*** |

**Significance level: ‘***’ 0.001, ‘**’ 0.01, ‘*’ 0.05, ‘~’ 0.1**

**Supplementary Table S3.** Sources of different climatic oscillations data.

| Climatic Oscillation | SOURCE |
| --- | --- |
| IOD | <https://psl.noaa.gov/gcos_wgsp/Timeseries/DMI/> |
| SIOD | <https://www.jamstec.go.jp/virtualearth/general/en/index.html> |
| MJO | <https://www.psl.noaa.gov/mjo/mjoindex/> |
| PDO | <https://psl.noaa.gov/data/correlation/pdo.data> |
